# Supplementary material for: Skeletal Deformities in Osterix-Cre;Tgfbr2f/f Mice May Cause Postnatal Death
Source: Genes (Basel). 2021 Jun 25;12(7):975. doi: 10.3390/genes12070975 (PMC8307487; doi:10.3390/genes12070975)
Supplement: Supplementary file 1 [file genes-12-00975-s001.zip › genes-1229354-supplementary.pdf]

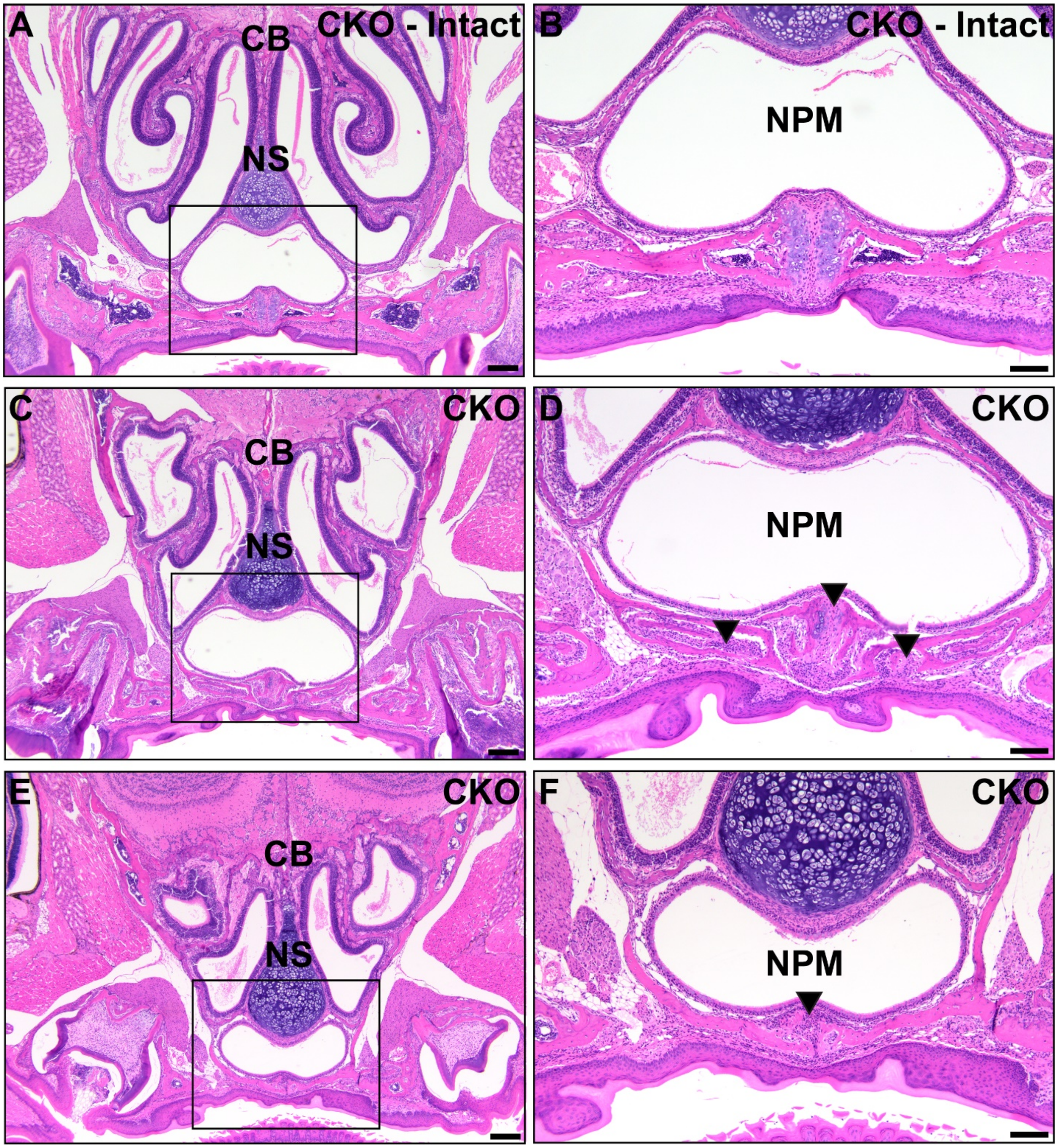

Figure S1. Bone Pathology of the Palate. Some CKO mice had appropriate, complete bone and cartilage structures forming the hard palate (CKO-Intact: A,B) while others (CKO: C,D and E,F) had variable bone dysplasia. In some affected CKO mice, bones were thin and scrolled with gaps in bones filled with fibrous connective tissue and an oval precursor population similar to that seen in the calvaria (C,D). In some CKO mice there was less than expected cartilage in this bone (D), while others were devoid of central cartilage (F). All assessed mice had continuous soft tissue ventral to the palate bone. Note the compressed nasopharyngeal structural anatomy with asymmetrical and smaller airspaces in affected CKO mice (C,E) and the dysplastic (C,E) and unerupted (E) molars. One HET mouse and 4 CKO mice had changes consistent with those shown here. Similar dysplastic changes were not present in Cre- mice (not shown). Scale bars A, C, E = 200 µm. Scale bars B, D, F = 100 mm. CB: Cribriform; NS: Nasal Septum; NPM: Nasopharyngeal meatus; Arrows: precursor cell population.

Incidence of Histopathological Changes in HET and CKO Mice				
	#/4 HET Affected	% HET Affected	#/9 CKO Affected	% CKO Affected
**Muscle Lesions**				
Myocardial Necrosis	2	50%	6	67%
Skeletal Muscle: Multinucleated	1	25%	6	67%
**Long Bone Lesions**				
Epiphysis Dysplasia	3	75%	9	100%
Long Bone Cortices/Proliferations	2	50%	6	67%
Articular Cartilage Dysplasia	3	75%	9	100%
**Other Bony Changes**				
Rib Dysplasia	1	25%	2	22%
Vertebra	4	100%	9	100%
Sternum Dysplasia	2	50%	8	89%
**Skull Lesions**				
Calvaria Dysplasia	3	75%	9	100%
Precursor Cells in Skull Sites	3	75%	8	89%
**Tooth & Oral Lesions**				
Molar Dysplasia	3	75%	9	100%
Unerupted Molars	2	50%	8	89%
Upper & Lower Molars Affected	1	25%	8	89%
Presence of Pocket/Teeth Absent	3	75%	7	78%
Oral Mucosal Epithelium Damage	3	75%	8	89%
Hard Palate Bone Dysplasia	1	25%	4	45%
**Nose & Olfactory Bulb Lesions**				
Ethmoid Turbinate Dysplasia	3	75%	9	100%
Nasal Septum Dysplasia	2	50%	7	78%
Cribriform Dysplasia	2	50%	7	78%
Olfactory Bulb Disorganization	1	25%	6	67%
Lateral Nasal Gland Dysplasia	3	75%	9	100%
Sinus Cell Debris/Inflammation	1	25%	5	56%
**Neutrophil Influx**				
Neutrophils at Turbinates	1	25%	7	78%
Neutrophils at Cribriform	2	50%	8	89%

Table S1. Incidence of histopathological lesions in HET and CKO mice. Comprehensive pathological evaluation of Cre-, HET and CKO mice revealed repeatable patterns of osteochondral dysplasia and other changes in HET and CKO mice. Four HET and nine CKO mice were assessed and the number and percent of mice exhibiting a given histopathological change are provided. None of the described lesions were present in the five examined Cre- mice, all of which had findings appropriate and consistent with postnatal mice in this age range.
